# An Integrative Bioinformatics Approach to Investigating TIMP3 and Immune Cell Infiltration: Prognostic and Clinicopathological Implications

**DOI:** 10.3390/ijms26188867

**Published:** 2025-09-11

**Authors:** Neelam Bhola, Chanchal Bareja, Amit K. Jaiswal, Daman Saluja

**Affiliations:** 1 Dr. B.R. Ambedkar Center for Biomedical Research, University of Delhi, Delhi 110007, India; neelambhola@gmail.com (N.B.); chanchal1862@gmail.com (C.B.); 2 School of Food Science and Environmental Health, Faculty of Sciences and Health, Technological University Dublin—City Campus, Central Quad, Grangegorman, D07 H6K8 Dublin, Ireland; amit.jaiswal@tudublin.ie; 3 Department of Allied and Basic Sciences, Shri Guru Gobind Singh Tricentenary University, Gurugram 122505, India

**Keywords:** TIMP3, bioinformatics analysis, immune infiltration, CRC, tumor microenvironment, biomarker

## Abstract

Tissue inhibitor of metalloproteinase 3 (TIMP3) serves as a prominent endogenous inhibitor of matrix metalloproteinases (MMPs), playing a crucial role in inhibiting metastasis, and angiogenesis. However, its exact contributions to colorectal cancer (CRC) remain largely unidentified. We aimed to ascertain the prognostic significance of TIMP3 in CRC patients through a bioinformatic approach. GEPIA, UALCAN, Kaplan–Meier plotter, LinkedOmics, cBioPortal, GeneMANIA, TIMER, TISIDB, the ScTIME database, TISMO, TIDE, CAMOIP, and TISCH2 were employed to comprehensively analyze the differential expression, prognostic value, genetic alterations, signaling pathways, immune cell infiltration, tumor microenvironment (TME) and associated genes of TIMP3 in CRC patients. Compared to adjacent normal tissues, we observed a significant downregulation of TIMP3 expression in CRC samples. Gene interaction networks elucidated that TIMP3 and its associated genes play a pivotal role in cancer progression, particularly in processes critical to colorectal cancer, such as extracellular matrix organization and angiogenesis. Analysis of the TME further indicates that TIMP3 expression was intricately associated with diverse immune cell types infiltration levels, chemokines, and immunomodulators. Most importantly, those with elevated TIMP3 expression had improved immunological scores. Moreover, TIMP3 exhibited strong correlations with major infiltration-related immune cells, including B cells, CD8^+^ T cells, CD4^+^ T cells, macrophages, neutrophils, dendritic cells, and fibroblasts. Furthermore, improved immunotherapeutic responses against PD-1/PD-L1 were linked to elevated TIMP3 levels. In TIMP3-high groups, there was a considerable increase in IL10, PDCD1, CD80, CXCL9, and CXCR3. This highlights the extensive influence of TIMP3 downregulation on the immune milieu within CRC. Our findings emphasize the multifaceted involvement of TIMP3 in CRC, not only influencing the molecular pathways associated with cancer progression, but also intricately shaping the immune microenvironment. As a result, TIMP3 appears promising as a potential CRC therapeutic target.

## 1. Introduction

Colorectal cancer is one of the most prevalent malignant tumors of the gastrointestinal tract due to its high morbidity and mortality rate [1]. Globally, colorectal cancer has the second-highest mortality rate but the third-highest incidence rate, with over 1.9 million new cases and approximately 935,000 deaths annually, ranking third in incidence and second in cancer-related mortality [2,3]. Recent advancements in surgical methods, chemotherapy, and the identification of molecular targets have improved the survival rates of CRC patients. Despite advances in screening and treatment, the overall five-year survival rate remains around 65.4%, with marked differences depending on the stage at diagnosis: 91.5% for localized disease, 74.6% for regional spread, and only 16.2% for metastatic cases [4]. Moreover, the growing emphasis on early detection has played a key role in further enhancing outcomes [5,6]. The pathogenesis of advanced CRC is intricately intertwined with genetic predisposition, lifestyle factors, and colorectal adenoma, resulting in poor prognosis [7]. Colonoscopy is regarded as the gold standard for CRC screening due to an outstanding specificity of over 95%. Unfortunately, it is a surgical procedure that requires intestinal preparation and can sometimes lead to catastrophic problems [8]. The lack of reliable prognostic indicators for early detection remains a significant hurdle, contributing to the suboptimal prognosis of CRC, despite the array of therapeutic options available [9]. Screening and surveillance strategies are essential for early detection and management of colorectal cancer and its associated pathologies [10]. Moreover, there is currently no well-established strategy for treating metastatic tumors that are not amenable to surgical removal, as these often show poor response rates to both chemotherapy and radiation therapy [11].

Maintenance of the extracellular matrix (ECM) and the integrity of healthy tissues is significantly influenced by the ratio of matrix metalloproteinases (MMPs) to tissue inhibitor of metalloproteinases (TIMPs) [12]. Disruption of the balance between MMP and TIMP function during cancer development could potentially worsen patient prognosis by affecting the invasion and spread of cancer cells [13]. TIMP3, one of the four members of TIMPs gene family, is located on chromosome 22q12.1-q13.2 [14]. TIMP3 is distinct from the other TIMPs in that it is a component of the extracellular matrix (ECM), in contrast to being soluble. TIMP3 is a naturally occurring angiogenesis inhibitor that suppresses the growth of malignancies by limiting the number of vessels in the vascular bed of the tumor [13,15,16].

In addition, TIMP3 has been shown to induce endothelial apoptosis and to directly interact with vascular endothelial growth factor (VEGF) receptor-2 to prevent angiogenesis and choroidal neovascularization [17,18]. In vitro and in vivo studies using a variety of tumor types have revealed that high TIMP3 expression induces apoptosis of tumor cells. TIMP3 can function as a tumor suppressor protein by influencing migration, invasion, and tumorigenicity [19,20,21]. While the clinical relevance of TIMP3 expression in malignant tumors has been extensively studied [22,23], its relationship with tumor-infiltrating lymphocytes and cancer prognosis remains unclear. The advancement of medical knowledge necessitates the investigation of CRC pathophysiology and the discovery of new therapeutic targets.

TIMP3, a member of the tissue inhibitors of metalloproteinases family, is vital for regulating immune infiltration and inhibiting tumor growth in multiple cancer types. In glioblastoma, elevated TIMP3 expression is associated with improved patient survival and enhanced infiltration of immune cells [24]. TIMP3 expression has been correlated with decreased protumor hematopoietic cell populations and reduced activity of gene sets associated with inflammation in papillary thyroid carcinoma [25]. Plasma levels of TIMP3 may also serve as a potential biomarker for monitoring the progression of oral cancer [26]. By decreasing IL-6 synthesis, TIMP3 overexpression increases osteosarcoma’s susceptibility to cisplatin [27]. Despite the fact that TIMP3 has been studied in a variety of malignancies, currently no comprehensive research exists that connects the expression levels of the TIMP3 gene to the prognosis of CRC patients or its relationship with the TME and immune infiltration.

With the rapid advancements in immune infiltration research and microarray technology, bioinformatics has become an essential approach for analyzing the expression patterns and prognostic relevance of TIMP3 gene in CRC. Integrating relevant databases and applying advanced technologies are key to conducting more comprehensive and insightful investigations.

This study entailed a thorough bioinformatics analysis of TIMP3 across numerous databases, with the goal of determining its clinical importance, prognostic implications, and expression patterns in CRC. While previous studies have examined the association of TIMP3 with prognosis and pathogenesis in cancer [28,29], including some links to immune processes, our work provides novel insights by integrating multi-omics data and single-cell transcriptomic analyses to specifically characterize the expression patterns of TIMP3 across diverse immune cell types within the tumor microenvironment. Web-based resources were significantly helpful in exploring the function of TIMP3 in transcriptional alterations, functional networks, and tumor immunity. The TIMER and GEPIA databases were used to evaluate TIMP3 mRNA expression in CRC, while the UALCAN database was employed to analyze TIMP3 protein levels. Using the TIMER and TISIDB databases, the relationship between TIMP3 expression and tumor-infiltrating immune cells (TIICs) in CRC was examined. This study also utilized the TIMER and GEPIA databases to investigate the correlation between TIMP3 expression and gene markers of TIICs. Further investigation of genes co-expressed with TIMP3 and their regulatory networks was carried out using Gene MANIA and LinkedOmics analysis. Genetic alterations and correlation analysis in CRC were conducted using the cBioPortal database. Based on these comprehensive analyses, novel molecular indicators within CRC’s microenvironment were identified, suggesting their potential significance in customized treatment approaches.

## 2. Results

### 2.1. TIMP3 is Highly Expressed in Normal Cells Compared to CRC

**Figure 1:**
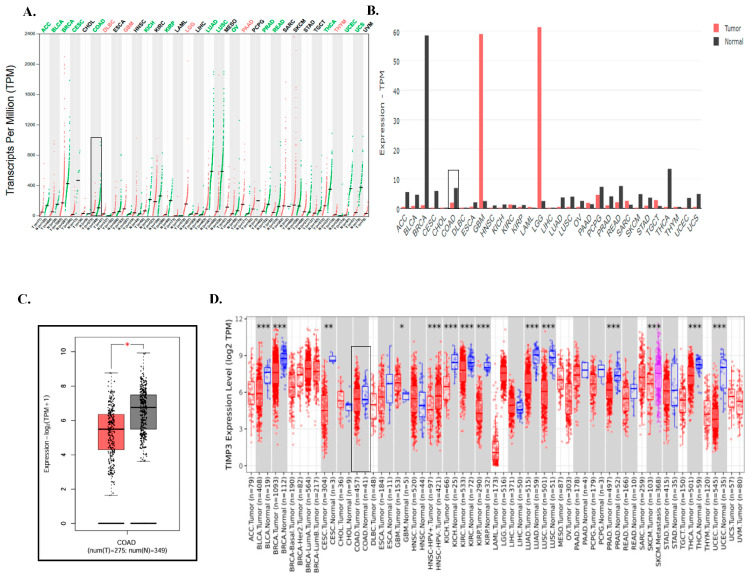
Expression levels of the TIMP3 gene in pan-cancer. (**A**–**C**) Expression profiles of the TIMP3 gene in different cancer types and paired normal tissue samples from the TCGA and GTEx datasets, respectively, from the GEPIA database. Red color indicates tumor tissue, and grey color indicates normal tissue samples. Black boxes highlight the COAD samples, to emphasize differential expression in that tissue compared to others while the short black horizontal lines within each group represent the median expression level of TIMP3 across tumor or normal samples. (**D**) Expression profiles of the TIMP3 gene in different cancer types and paired normal tissue samples from the TIMER database. The data for colorectal cancer is highlighted by adding a box around it. Statistical significance was calculated with the Wilcoxon test. * *p* < 0.05; ** *p* < 0.01; *** *p* < 0.001.

We determined the differential expression of the TIMP3 gene in matched normal tissues and different types of malignancies (pan-cancer) through an online search of the GEPIA database (Figure 1A,B). In comparison to the equivalent normal tissues, TIMP3 was significantly reduced in various cancer types, including lung adenocarcinoma (LUAD), kidney chromophobe (KICH), lung squamous cell carcinoma (LUSC), colorectal cancer (CRC), and uterine carcinosarcoma (UCS) tissues (Figure 1B). Glioblastoma multiforme (GBM) and lower-grade glioma (LGG) exhibited markedly higher TIMP3 expression compared to their corresponding normal tissues, in contrast to most other tumor types, where TIMP3 expression was lower than in adjacent normal tissues. This differential pattern suggests a possible tissue- or context-specific regulatory mechanism of TIMP3 in gliomas, which may warrant further investigation.

Analysis of the data using the GEPIA 2.0 tool indicated a statistically significant decrease in TIMP3 gene expression in CRC (*p* ≤ 0.05), as shown in Figure 1C. Furthermore, utilizing TIMER2.0, we examined TIMP3 expression levels across all TCGA cancer types. The above finding was corroborated by the TIMER database, which also showed decreased expression of TIMP3 in various tumor tissues in comparison to the matched normal tissue (Figure 1D).

**Figure 2:**
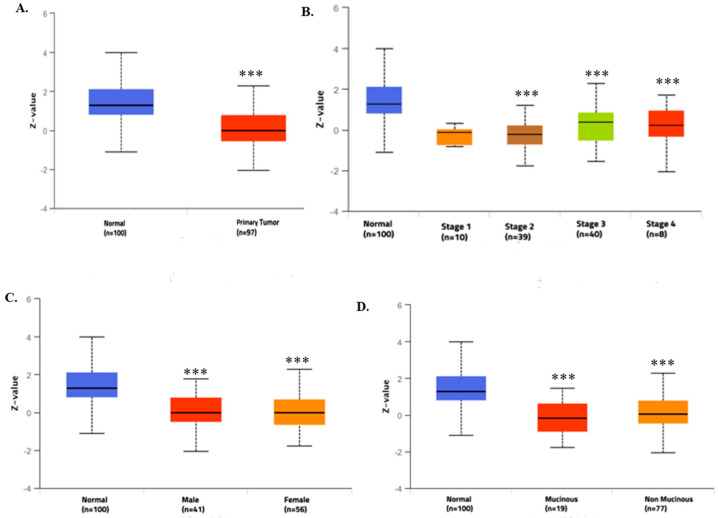
TIMP3 protein expression level in COAD patients. (**A**) Boxplot showing relative TIMP3 protein levels in normal individuals and COAD patients, from UALCAN. (**B**–**D**) TIMP3 relative expression in colon cancer samples compared with normal samples and analyzed by cancer stages, gender, and tumor histological subtype. *** *p* < 0.001.

To strengthen our understanding of the role of TIMP3 in CRC, we utilized TCGA data from UALCAN to assess TIMP3 protein expression and its association with the clinicopathological characteristics of CRC. The analysis revealed a significant decrease in TIMP3 protein expression in CRC samples compared to normal samples (*p* ≤ 0.001), as illustrated in Figure 2A. Subsequent subgroup analyses based on cancer stages, gender, and histological subtypes consistently showed lower TIMP3 protein levels in CRC tissues relative to normal tissues (Figure 2B–D). Specifically, across different cancer stages, TIMP3 expression was notably lower in colorectal cancer compared to normal tissues, demonstrating statistical significance (*p* ≤ 0.001), as depicted in Figure 2B. Additionally, male and female tissues did not exhibit any difference in TIMP3 expression levels in COAD patients (Figure 2C). Furthermore, TIMP3 expression diminished in both mucinous and non-mucinous histological subtypes of COAD compared to their respective normal counterparts (Figure 2D). These findings underscore a consistent reduction in TIMP3 mRNA levels in CRC across various datasets. Thus, the diminished expression of TIMP3 in these cancer patients suggests its potential significance in studying invasion and metastasis, given its crucial role in maintaining extracellular matrix (ECM) homeostasis and inhibiting metastatic processes.

### 2.2. The Immune Response in CRC Tissues Is Enhanced by TIMP3 and Its Co-Expressed Genes

**Figure 3:**
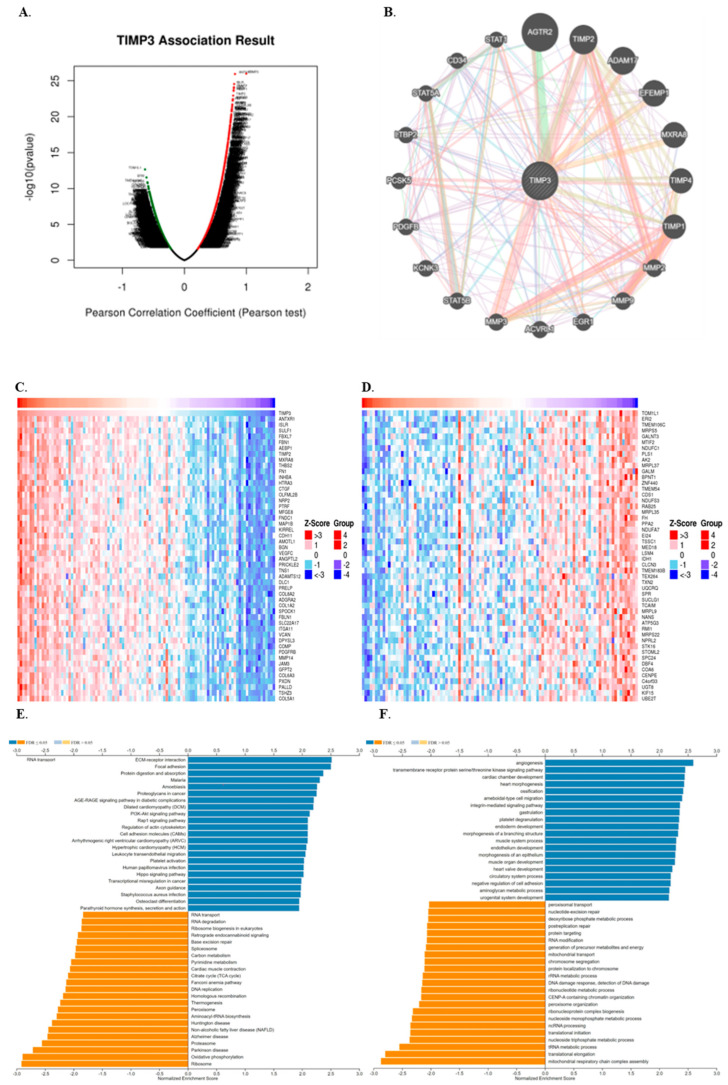
Genes that are differentially expressed in relation to TIMP3 and associated functional enrichment analysis, as determined via the LinkedOmics database and GeneMANIA. (**A**) volcano map showing the relationship between TIMP3 and the genes that are expressed differently in COAD, as determined by *t*-test. (**B**) PPI network of TIMP3 with its interactive genes from the GeneMANIA database. (**C**,**D**) Heatmaps displaying positive and negative correlations between genes and TIMP3 in COAD. Genes with positive correlations are shown in red, and those with negative correlations are shown in blue. (**E**) KEGG pathway analysis (**F**) Biological processes.

Following an analysis of TIMP3 expression and its prognostic implications in COAD patients, using LinkedOmics, we identified co-expressed genes and associated pathways to explore its potential biological roles in colon cancer. LinkedOmics’ volcano plot (Figure 3A) displayed 13,482 genes that were associated with TIMP3. Among these, 6249 genes were positively correlated, while 7233 genes were negatively correlated. Red dots exhibit a significant positive correlation with TIMP3, whereas green dots indicate a significant negative correlation (false discovery rate, FDR ≤ 0.01). We next examined 20 co-expressed genes that were significantly correlated with TIMP3 using the GeneMANIA “co-expression” module to gain insight into TIMP3’s molecular mechanism in carcinogenesis (Figure 3B). AGTR2, TIMP2, ADAM17, EFEMP1, MXRA8, TIMP4, TIMP1, MMP2, MMP9, EGR1, ACVRL1, MMP3, STST5B, KCNK3, PDGFB, PCSK5, LTBP2, STAT5A, CD34, and STAT1 were the top 20 proteins predicted to interact with TIMP3. Additional pathway enrichment studies were performed using GeneMANIA. The top 50 genes are shown in the heat map (Figure 3C,D). Notably, TIMP3-related genes were directly involved in ECM receptor interaction, focal adhesion, protein digestion and absorption, and amoebiasis (Figure 3E). Many of these genes belong to families involved in extracellular matrix (ECM) organization, cell adhesion, and tissue remodeling, which align with TIMP3’s known role as an inhibitor of matrix metalloproteinases (MMPs). The most enriched biological terms were angiogenesis, transmembrane protein transfer serine/threonine kinase signaling pathway, amoeboid-type cell migration, and heart morphogenesis (Figure 3F).

### 2.3. TIMP3 Displays an Intricate Association with Immune-Related Regulatory Molecules

**Figure 4:**
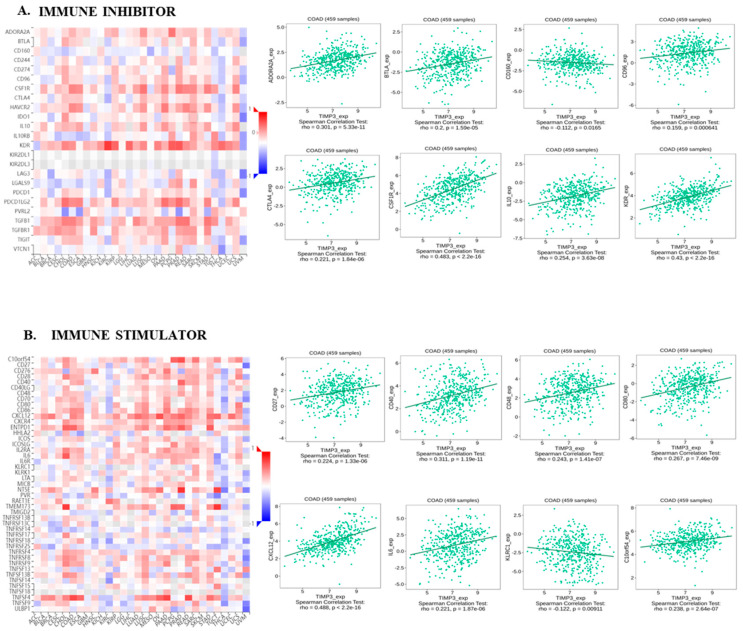
Correlation analysis of TIMP3 expression with immunomodulators in COAD from the TISIDB database. (**A**) Heatmap of the correlation between TIMP3 and immunoinhibitors across different cancer samples from TISIDB. (**B**) Relationships between TIMP3 and immunostimulators in COAD. *p* values and partial correlation were obtained by Spearman’s rank correlation test after adjusting by purity. All of the data was displayed as heatmaps and scatterplots.

**Figure 5:**
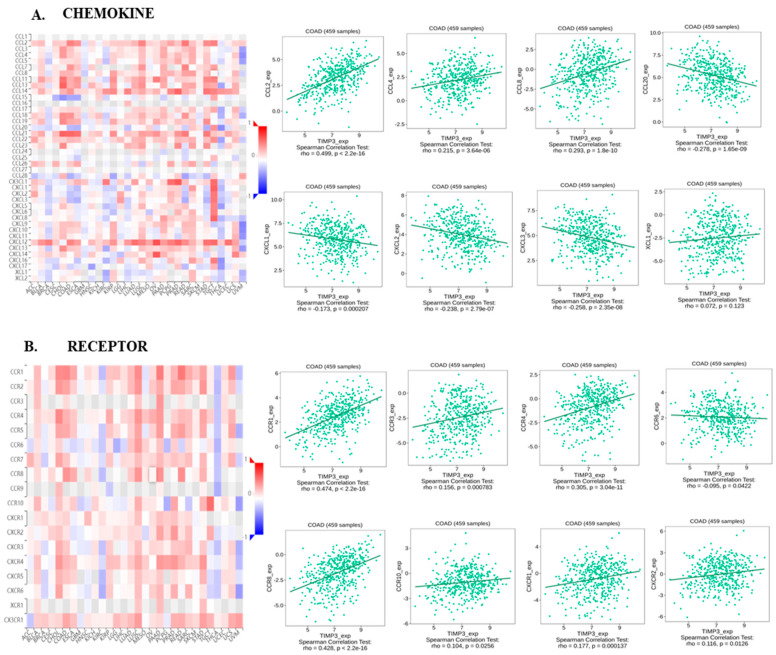
Correlation analysis of TIMP3 expression with chemokines and receptors in COAD from the TISIDB database. (**A**) Heatmap of the correlation between TIMP3 and chemokines across different cancer samples from TISIDB. (**B**) Relationship between TIMP3 and receptors in COAD. *p* values and partial correlation were obtained by Spearman’s rank correlation test after adjusting by purity. All of the data was displayed as heatmaps and scatterplots.

We next explored the intricate connections between TIMP3 and various components such as chemokines, receptors, immune inhibitors, and stimulators using the comprehensive resources of the TISIDB database. This in-depth analysis aimed to enhance our comprehension of the intricate interplay between TIMP3 expression and immune infiltration. According to our findings, multiple immunoinhibitors and immunostimulators showed positive associations with the levels of TIMP3 in COAD, including ADORA1, BTLA, CD96, KDR, IL10, CD27, CD40, and CXCL12. In COAD, TIMP3 was negatively correlated with multiple immune stimulators and immunoinhibitors, including CD160 and KLRC1 (Figure 4A,B). Immune cell activation and recruitment are significantly influenced by chemokines and chemokine receptors. Using TISIDB, correlations between chemokines and chemokine receptors with TIMP3 expression were also assessed. CXCL1, CXCL2, CXCL3, and CCR6 were among the chemokines with which TIMP3 had a negative correlation, while CCR6 was a chemokine receptor which showed negative correlation with TIMP3 expression (Figure 5A,B). Taken together, these results suggest that TIMP3 plays a role in immune cell infiltration in COAD and may be useful in anticipating the efficacy of immunotherapy, since it is closely associated with immune cell infiltration.

### 2.4. Genetic Alteration of TIMP3 Is Significantly Associated with Survival of CRC Patients

**Figure 6:**
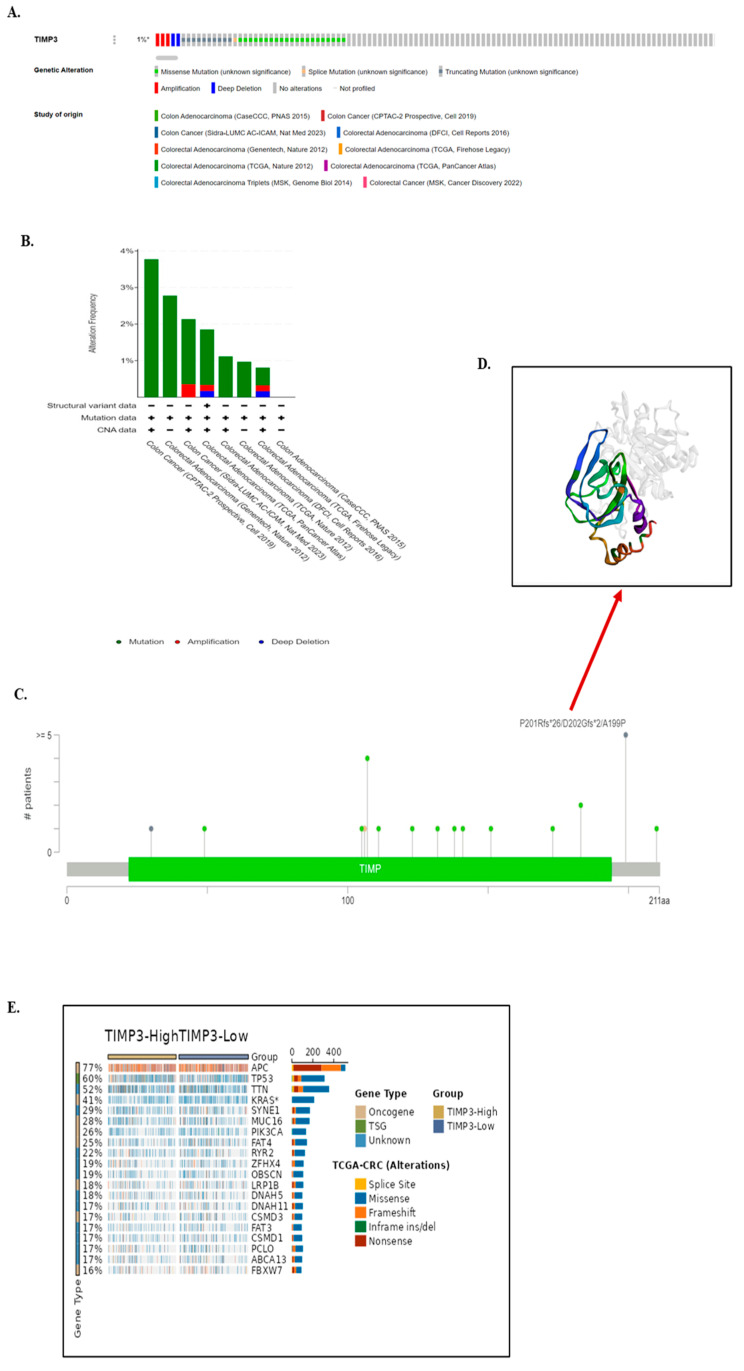
Five TIMP3 mutation features found in various TCGA cancers. We examined the mutation characteristics of TIMP3 for TCGA tumors using the cBioPortal program. (**A**–**C**) The mutation frequency, together with the mutation type and mutation site, are displayed. Distribution of TIMP3 alterations (missense, splice, truncating mutations, amplification, and deep deletion) across multiple CRC cohorts. Frequency of TIMP3 alterations across individual studies (**D**) The 3D structure of TIMP3 highlights the mutation site with the highest frequency of change, corresponding to P201Rfs*/D202Gfs*/A199P. The asterisk (*) denotes a premature stop codon generated by frameshift mutations, leading to truncated TIMP3 protein products. (**E**) According to the CAMOIP database, distinct somatic mutations were identified in TCGA-CRC. Heatmap showing co-occurring genetic alterations in key oncogenes and tumor suppressor genes between TIMP3-high and TIMP3-low colorectal cancer groups. Colors indicate alteration types: green, missense; red, truncating (frameshift, nonsense, splice); blue, in-frame mutation; purple, fusion; deep red, amplification; light blue, deep deletion; grey, no alteration.

Genetic alterations of *TIMP3* in CRC cancer samples were also studied utilizing the cBioPortal database. The database queried for *TIMP3* gene mutations in 2850 samples from 10 cancer studies of CRC. The gene set or pathway was altered in 1% of the queried samples, with a somatic mutation frequency of 1.1%. In patients with multiple mutations, sites were located within 1 and 211 amino acids of the TIMP3 propeptide and *TIMP3* domain. In total, 32 mutations were reported, among which 21 are missense mutations, 10 are truncating mutations, and 1 is a splice mutation (Figure 6A,B). We also observed that frameshift and missense mutations were the major types of genetic alterations in TIMP3, among which the P201Rfs*26/D202Gfs*2/A199P alteration was detected in ten cases of CRC. (Figure 6C,D). We used the CAMOIP database to identify the top 20 mutated genes in the TCGA-CRC datasets in order to investigate the mutational landscape of various TIMP3 expression groups. In both of these categories, the most often mutated genes are APC, TP53, and TTN. Among these, there were notable differences in the mutation frequencies of the KRAS and PIK3CA genes in TCGA-CRC between the groups with high and low TIMP3 expression. The mutation burden may be slightly higher in the TIMP3-low group for several genes (Figure 6E).

**Figure 7:**
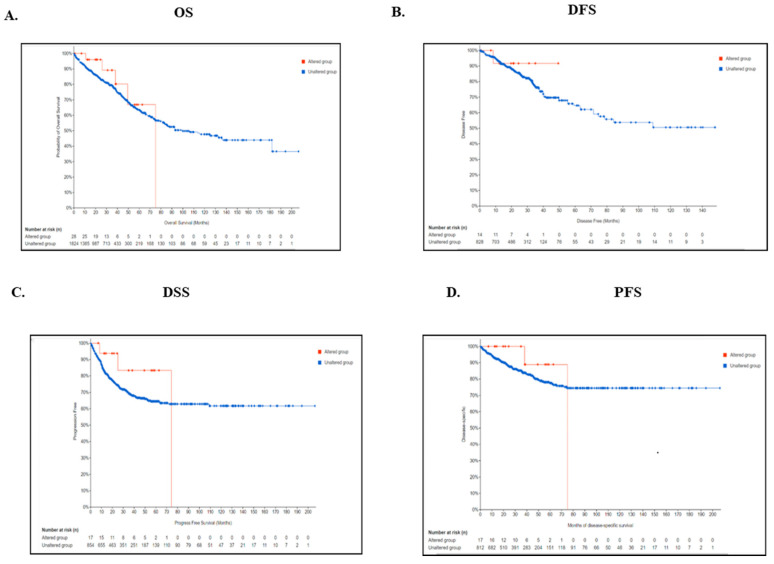
(**A**–**D**) Relationship between mutation status and CRC overall survival, disease-free survival, disease-specific survival, and progression-free survival based on cBioPortal.

We also looked into the possibility that patient survival in colorectal cancer was related to variations in the TIMP3 gene. Nevertheless, our findings suggest that the genetic alterations were not associated with poorer overall survival (OS) (*p* = 0.221), disease-free survival (DFS) (*p* = 0.355), progression-free survival (*p* = 0.924), or disease-specific survival (*p* = 0.231) in patients with colorectal cancer (Figure 7A–D).

### 2.5. Single-Cell Expression Analysis Highlights TIMP3’s Role in TME Remodeling and Immune Regulation

**Figure 8:**
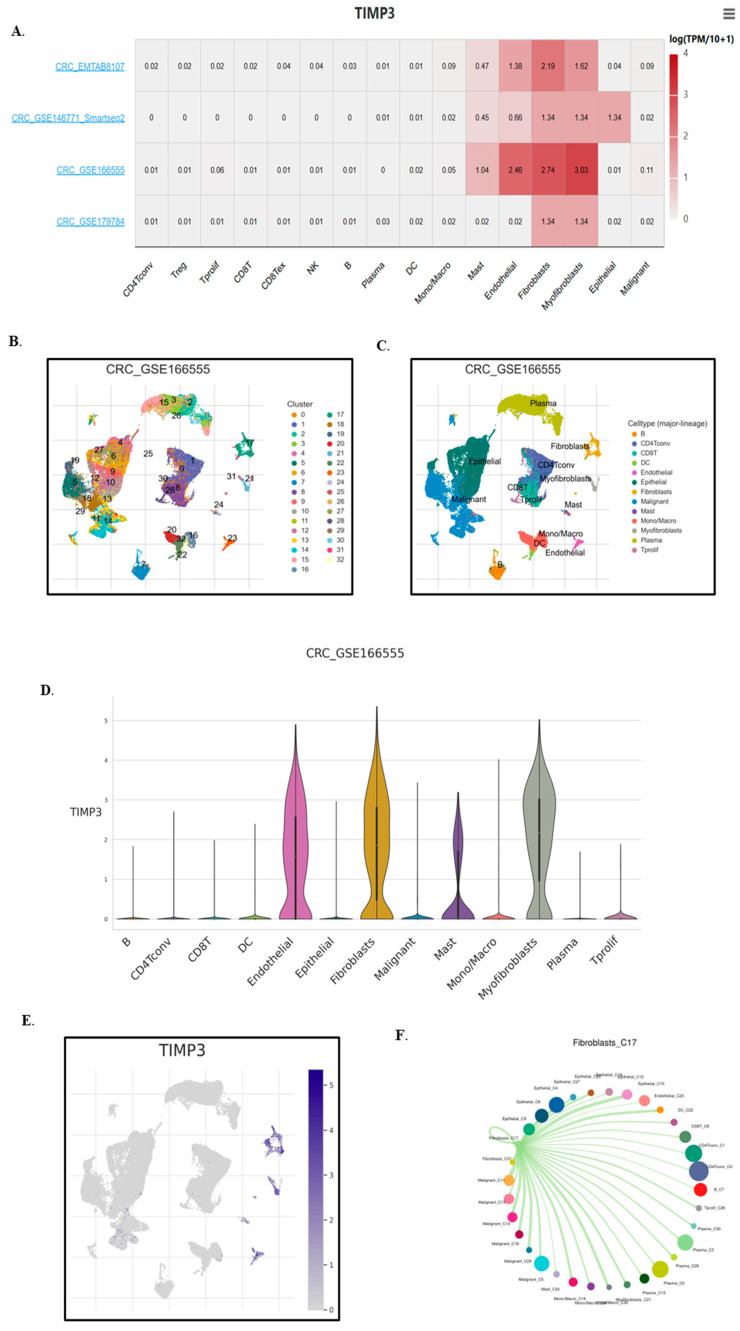

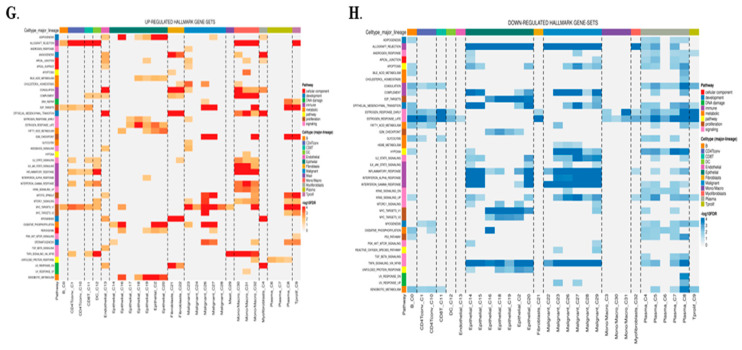
Expression analysis of TIMP3 in the TME, based on a single-cell RNA-seq database. (**A**) The distribution of TIMP3 expression in various datasets downloaded from TISCH2. (**B**–**E**) The expression of TIMP3 in various TME cells in dataset CRC_GSE166555, visualized by UMAP and violin plot. The cell types are colored according to the major annotations. (**F**) Cell–cell communication plot. (**G**) The heatmap depicted functionally enriched, upregulated hallmark pathways, identified through differentially expressed genes in each cell type within the GSE166555 dataset. (**H**) The heatmap depicted functionally enriched, downregulated hallmark pathways, identified through differentially expressed genes in each cell type within the GSE166555 dataset.

We analyzed the CRC_EMTAB8107, CRC_GSE146771, CRC_GSE166555 and CRC_GSE179784 colorectal cancer datasets to investigate TIMP3 expression across different cell populations within the TME. The results showed that TIMP3 expression was broadly distributed throughout a variety of immune cell types, including B cells, conventional CD4^+^ T (CD4Tconv) cells, CD8^+^ T cells, and monocytes. In addition to immune cells, high expression of TIMP3 was also observed in macrophages, mast cells, endothelial cells, fibroblasts, myofibroblasts, and epithelial cells, as confirmed by single-cell sequencing (Figure 8A). A thorough analysis of the CRC_GSE166555 dataset from the TISCH2 database provided insights into the expression patterns of TIMP3 at the single-cell level. The dataset revealed 32 distinctive cell clusters, the majority of which were made up of immune cells (Figure 8B) and thirteen different cell types (Figure 8C). TIMP3 expression is primarily observed in endothelial cells, fibroblasts, mast cells, and myofibroblasts, with relatively low expression in lymphoid and other immune cells (Figure 8D,E). Bulk transcriptomic analyses revealed statistical associations between TIMP3 expression and immune infiltration scores. However, single-cell RNA-sequencing data demonstrated that TIMP3 expression is primarily restricted to stromal populations, including endothelial cells, fibroblasts, and myofibroblasts, rather than to lymphoid or myeloid immune cell subsets (Figure 8D). These findings suggest that the observed associations are more likely mediated indirectly, for example through extracellular matrix remodeling or vascular modulation, rather than by direct TIMP3 production from infiltrating immune cells. Accordingly, we avoid attributing a direct and widespread role for TIMP3 within lymphoid compartments and recommend further validation, such as immunohistochemistry or analyses of independent single-cell RNA-sequencing datasets, to clarify its potential influence on immunotherapy responsiveness via stromal pathways.

The widespread expression of TIMP3 in almost every kind of immune cell suggests that it plays a critical role in regulating immune suppression in the TME. This might involve suppressing immune cell activation, encouraging immune inhibitory cell growth and activity, and altering signaling pathways linked to immune evasion. Among all cell types, fibroblast cells had the highest expression of TIMP3; therefore, cell communication analysis was performed to understand its relevance. These results indicated strong interactions between fibroblast cells and monocytes/macrophages, natural killer (NK) cells, CD4^+^ T cells, and CD8^+^ T cells (Figure 8F). Gene set enrichment analysis showed that fibroblast cells were positively associated with upregulated HALLMARK pathways, including angiogenesis, apoptosis, coagulation, cholesterol homeostasis, complement system, epithelial mesenchymal transition, IL2-STAT-5 signaling, myogenesis, and the UV response (Figure 8G). In contrast, fibroblast cells were negatively associated with the early and late estrogen response (Figure 8H).

### 2.6. TIMP3 Modulates Immune Outcomes in the Tumor Microenvironment at a Single-Cell Level

**Figure 9:**
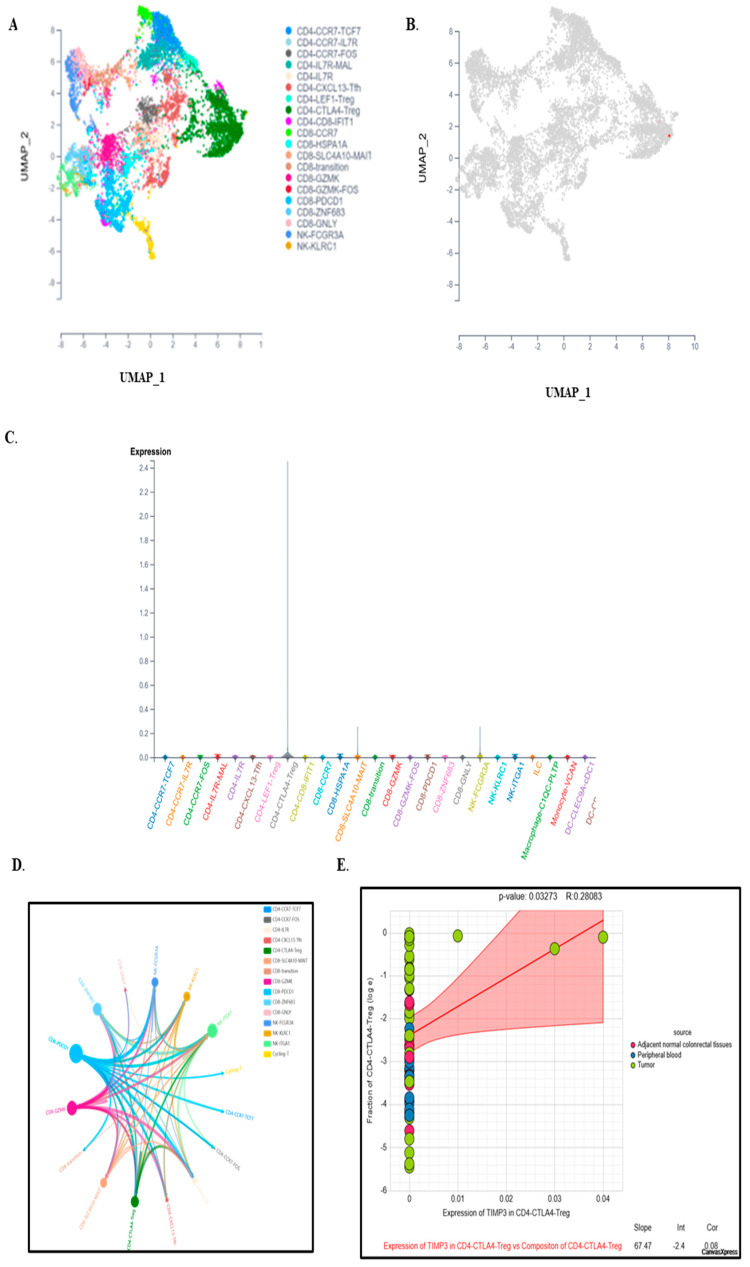
Relationship between the TIMP3 gene and the tumor immune microenvironment (TIME) at a single-cell level. (**A**) Different cell clusters in CRC based on scTIME. (**B**) The main cell clusters that TIMP3 is expressed in in CRC. (**C**) Expression of TIMP3 in different immune cells, according to the scTIME database. (**D**) Correlation between TIMP3 and immune cell infiltration. (**E**) Expression of TIMP3 in CD4-CTLA4-Treg cells in colorectal cancer.

Twenty primary cell types were identified from the cell populations (Figure 9A). According to the results, TIMP3 was mostly expressed by CD4-CTLA4-Treg cells (Figure 9B,C). TIMP3 expression is much higher in CD4^+^ CTLA4+ Tregs than in other T cell subsets. This emphasizes cell-type specificity, implying that TIMP3 has a distinct functional role in this Treg subpopulation. TIMP3 expression is positively correlated (R = 0.28, *p* = 0.0327) with the number of CD4^+^ CTLA4+ Tregs. This suggests that, when TIMP3 expression increases, so does the proportion of these Tregs, especially in tumor tissue relative to normal or peripheral samples.

In CRC, the circle plots revealed that CD8-PDCD1 and NK FC3RGA cells interacted with other CD4-CTLA4-Treg cells the most, demonstrating their active biological connections (Figure 9D). CD4-CTLA4-Treg cells also showed a positive correlation with TIMP3 expression, as shown in Figure 9E (*p* value = 0.03273, rho value = 0.28083). These results suggest that TIMP3 in CD8-PDCD1, CD4-CTLA4-Treg, and NK-FC3RGA cells may influence cell–cell interactions and associated immune cell abundance, which may alter TME immunological activity. 

### 2.7. Immune Cell Infiltration and TIMP3 in Patients with CRC

**Figure 10:**
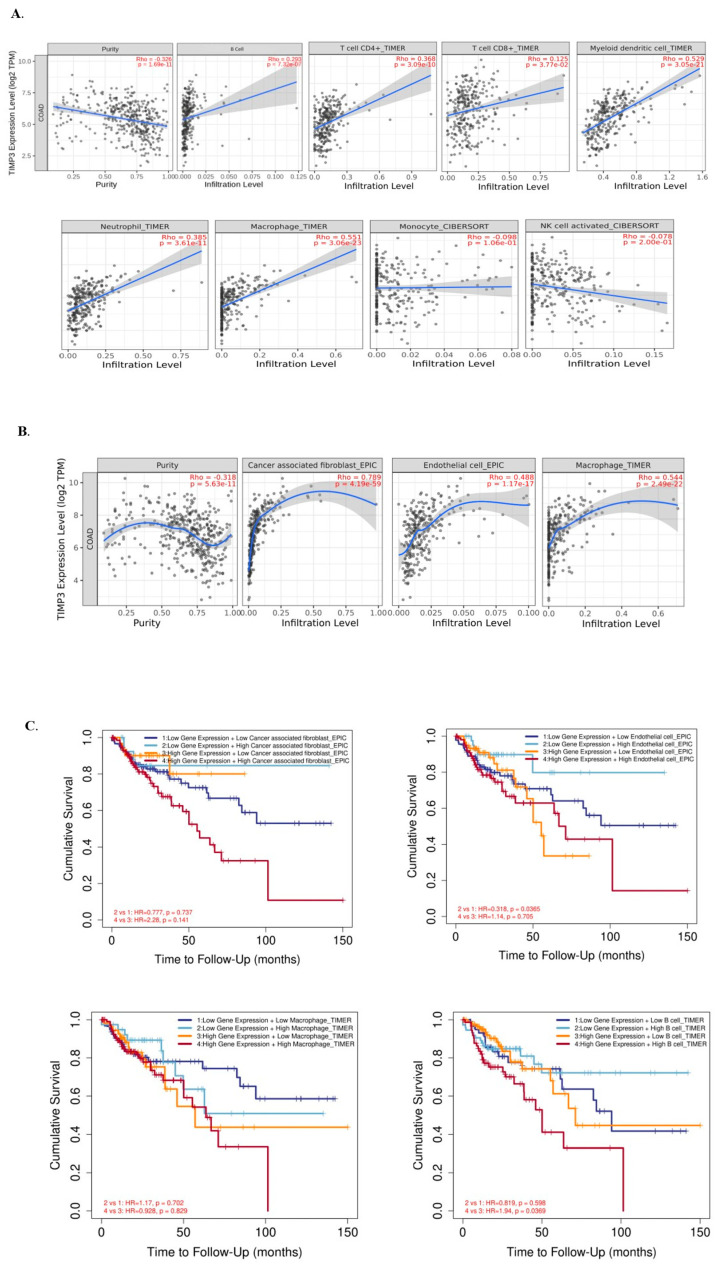
Correlation between TIMP3 expression and immune cell infiltration in COAD, as determined using the TIMER database. (**A**,**B**) Scatterplot showing the correlation between TIMP3 expression and B cells, CD4^+^ cells, CD8^+^ cells, myeloid dendritic cells, neutrophils, macrophages, monocytes, NK cells, fibroblasts, and endothelial cells among COAD samples. (**C**) KM survival plot stratifying COAD patients based on the expression of TIMP3 and immune cells.

The onset and progression of cancer are significantly influenced by the tumor microenvironment (TME) and its interactions with cancer cells [30]. The TIMER database was utilized to ascertain if the degree of immune cell infiltration in COAD was associated with TIMP3 expression. For this, we looked into the potential correlation between TIMP3 and the eight primary immune cells that infiltrate COAD (B cells, CD4^+^T cells, CD8^+^ T cells, myeloid dendritic cells, neutrophils, macrophages, monocytes, and NK activated cells) using the TIMER database. The database indicated a negative correlation between TIMP3 and tumor purity (r = −0.326, *p* = 1.69 × 10^–11^. We observed a significant positive correlation between TIMP3 and CD4^+^ T cells (rho = 0.368, *p* = 3.09 × 10^–10^), CD^8+^ T cells (rho = 0.125, *p* = 3.77 × 10^–2^), myeloid cells (rho = 0.529, *p* = 3.05 × 10^–21^), neutrophils (rho = 0.385, *p* = 3.61 × 10^–11^), macrophages (rho = 0.551, *p* = 3.06 × 10^–23^), and B cells ((rho = 0.293, *p* = 7.32 × 10^–7^). In contrast, we observed a negative correlation between TIMP3 and monocytes (rho = 0.098, *p* = 1.06 × 10^–1^) and NK cells (rho = 0.078, *p* = 2.00 × 10^–1^) (Figure 10A).

Using the gene module feature of TIMER3.0, we further explored how the expression of TIMP3 correlates with the degree of immune infiltration in COAD patients. Using the gene module feature of TIMER3.0, we further analyzed the correlation between TIMP3 expression and immune cell infiltration in COAD patients. TIMP3 expression showed significant elevation in fibroblasts, macrophages, and endothelial cells across multiple CRC datasets. A similar trend was also found after correlation analysis: TIMP3 was found to be positively corelated with fibroblasts, macrophages, and endothelial cells (Figure 10B). Stagewise comparison between different groups with high and low expression of TIMP3 and immune cells was performed using KM Plotter to predict the clinical relevance of TIMP3 using the immune outcome module of TIMER 3.0. The infiltration level is divided into low and high levels. The hazard ratio and *p* value for the Cox model and the log-rank *p* value for the KM curve is shown on the KM curve plot. High fibroblast and endothelial cell levels alongside high TIMP3 expression led to low overall survival in COAD patients. Furthermore, high TIMP3 expression with high macrophage levels leads to low overall survival and worse prognosis (Figure 10C).

### 2.8. TIMP3 Affects the Immune Microenvironment of CRC Tissues in TIMP3^High^ and TIMP3 ^Low^ Groups, and the Potential Role of TIMP3 in CRC

**Figure 11:**
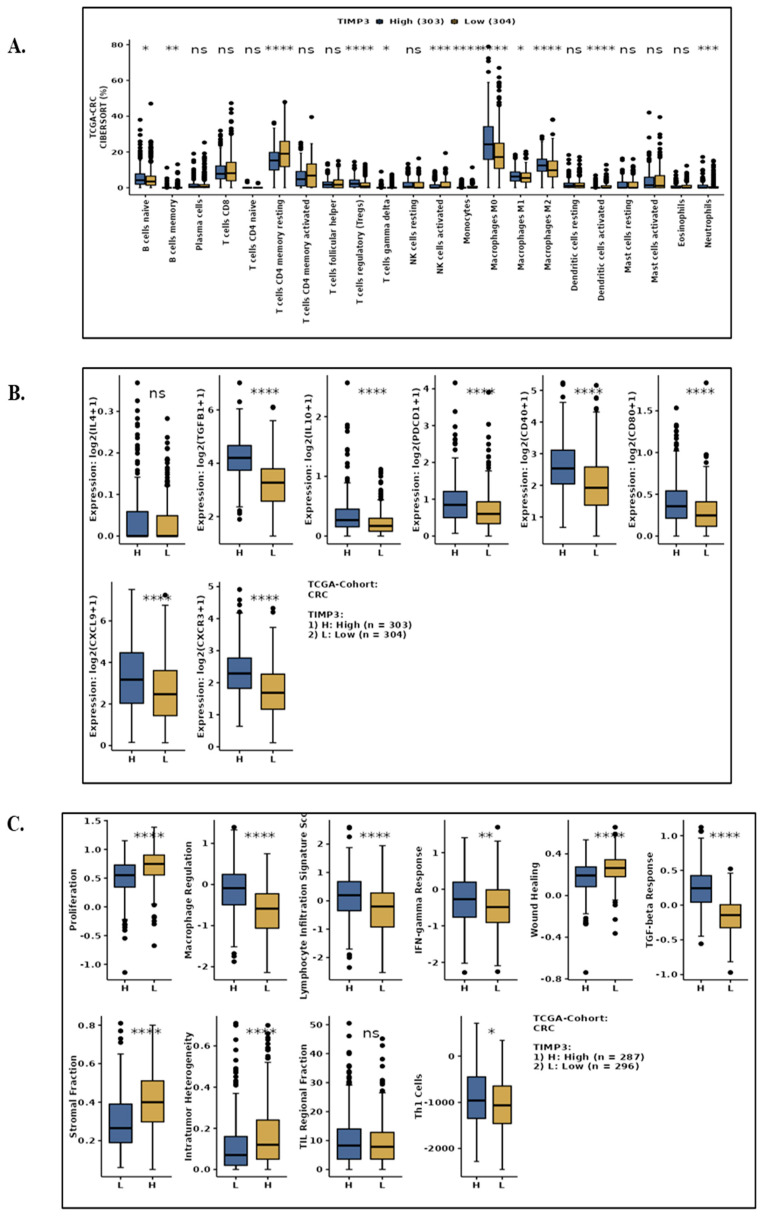

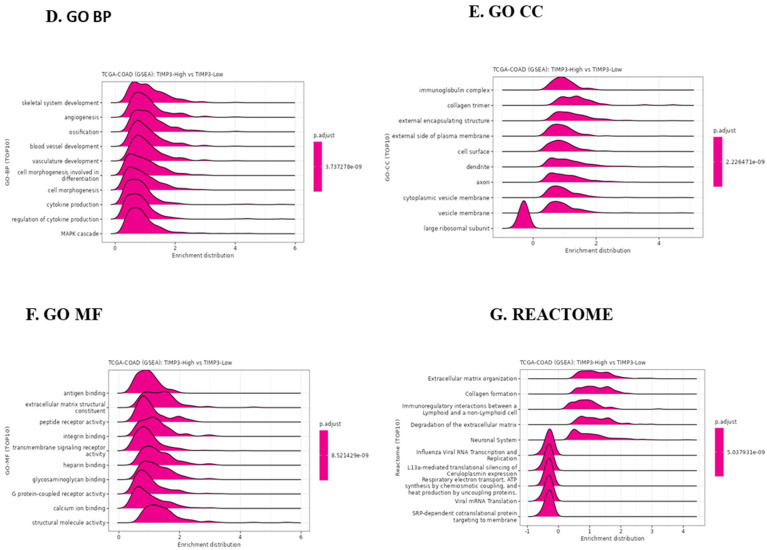
The relative abundance of 22 immune cell types was estimated using CIBERSORTx in TCGA colorectal cancer samples (TIMP3-high: n = 303, TIMP3-low: n = 304). (**A**) Boxplots show the distribution of each immune cell type as a percentage of the total infiltrate. Significant differences were observed in multiple immune populations between the groups. (**B**) Gene expression levels of the immune markers genes in colorectal cancer tissues with high TIMP3 or low TIMP3 expression, as determined from the CAMOIP database. (**C**) Immune scores, including the proliferation, macrophage regulation, the lymphocyte infiltration signature score, IFN-gamma response, wound healing, TGF-beta response, stromal fraction, intratumor heterogeneity, TIL regional fraction, and Th1 cells in patients with high TIMP3 expression and low TIMP3 expression in CRC. (**D**–**G**) Ridge plot from gene set enrichment analysis (GSEA) comparing high vs. low TIMP3 expression in GO-BP, GO-CC, GO-MF, and Reactome from TCGA-CRC data. Significance is denoted as follows: * *p* < 0.05, ** *p* < 0.01, *** *p* < 0.001, **** *p* < 0.0001; ns = not significant.

To explore the influence of TIMP3 on the TME, CAMOIP was used to detect the infiltrated proportions of immune cells using TCGA-CRC data. The variations in the 22 different TIIC types between TIMP3^high^ and TIMP3^low^ CRC patients were compared using TCGA-CRC CIBERSORT data. According to the data, TIMP^low^ had higher levels of CD8^+^ T cells, resting CD4^+^ memory T cells, follicular helper T cells, and resting mast cells. This data suggests a more inflammatory and immunosuppressive tumor microenvironment. In contrast, TIMP3^high^ tumors were enriched in monocytes, M1/M2 macrophages, naive B cells, and gamma delta T cells, indicating a more structured or differentiated immune landscape (Figure 11A). The decline in the M2 macrophage population in the TIMP3^high^ group was indirectly reflected in CRC patients. Earlier studies have also shown that tumor-associated macrophages in colorectal cancer have pro-inflammatory and anti-tumorigenic properties that promote anti-tumor T cell responses [31]. We also checked the population of certain immune marker genes in the TIMP3^high^ and TIMP3^Low^ groups and found that TGFβ1, IL10, PDCD1, CD80, CXCL9, and CXCR3 were all significantly enhanced in the TIMP3^high^ group (Figure 11B).

We next evaluated the immunological score of CRC patients with differential TIMP3 expression, since TIMP3 can influence the TME in CRC, particularly fibroblasts, macrophages, and endothelial-associated cells. When compared to TIMP3^low^ CRC patients, TIMP3^high^ CRC patients had significantly higher levels of immunological score indicators such as macrophage regulation, the lymphocyte infiltration signature score, the IFN-gamma response, the TGF-beta response, and Th1 cells (Figure 10C). Conversely, TIMP3^high^ CRC patients showed a significant decline in intratumor heterogenicity score (Figure 11C). After immunotherapy, a high immunological score is said to be linked to a favorable prognosis.

We used a GSEA enrichment approach to analyze the top 20 enriched GO-BP, GO-CC, GO-MF, and Reactome terms in the CAMOIP database in order to better understand the potential function of TIMP3 in CRC. Angiogenesis, blood/vascular development, MAPK cascade and cytokine signaling, and cell morphogenesis/differentiation are anticipated to be enriched by TIMP3-Low. The prevalence of mutations in oncogenic drivers is greater in TIMP3-low cancers. Angiogenesis, inflammation, and MAPK signaling pathway enrichment were seen in the TIMP3^low^ group (Figure 11D). This is consistent with a concept in which TIMP3 suppresses aggressive tumor behavior through ECM control, and its depletion encourages an inflammatory, angiogenic, and pro-invasive milieu.

The findings also indicated that immunoglobulin complex, collagen trimer, and the external encapsulating structure of the plasma membrane were the primary GO-CC terms (Figure 11E), and immune response and cytokine production were the primary GO-MF terms (Figure 11F). More immune-related terms (e.g., antigen binding), angiogenesis, and vasculature development were enriched in the TIMP3-high group compared to the TIMP3-low group, suggestive of a more aggressive, inflammatory, and invasive phenotype of TIMP3-high group. The primary Reactome terms involved ECM organization, collagen formation, and immunoregulatory interactions between lymphoid and non-lymphoid cells (Figure 11G). According to the functional enrichment analysis, TIMP3 in CRC was strongly linked to a number of relevant immunological pathways.

### 2.9. Predictive Value of TIMP3 in Modulating Immunotherapy

**Figure 12:**
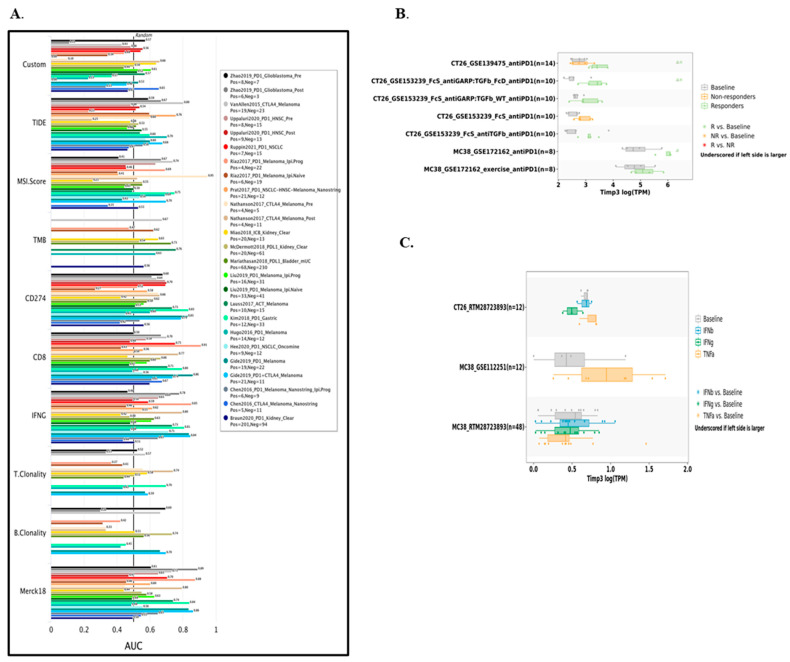
The effectiveness of CRC immunotherapy may be significantly impacted by TIMP3. (**A**) Predictive values of TIMP3 obtained from the TIDE database and comparison of TIMP3 and other published biomarkers for predicting immunotherapy response. (**B**) Disparities in TIMP3 expression between responder and non-responder mice, as well as before and after ICB treatment with anti-PD1, obtained from the TISMO database. (**C**) TIMP3 expression in tumor cell lines with cytokine treatment based on the TIMSO database.

Using survival outcomes and treatment response based on human immunotherapy cohorts, we evaluated the efficacy of TIMP3 in predicting response to immune checkpoint blockade (ICB) therapy in the TIDE database in comparison to other canonical biomarkers, such as Custom, Merck 18, B clonality, T clonality, IFNG, CD8, CD274, TMB, MSI score, and TIDE. According to Figure 12A, TIMP3 alone demonstrated area under curve (AUC) values over 0.5 in more than 15 out of 25 immunotherapy groups, which is comparable to the MSI score, indicating that it is a robust biomarker for cancer prediction. With an AUC above 0.5 in 9, 10, 11, 17, and 19 immunotherapy cohorts, respectively, TIMP3 showed more predictive value than Custom, TIDE, MSI SCORE, TMB, and CD274. Nevertheless, IFNG, CD8, T Clonity, B Clonity, and Merck18 showed AUC values over 0.5 in 7, 7, 5, 13, and 2 immunotherapy cohorts, respectively, outperforming TIMP3 in terms of predictive power.

Additionally, we used TISMO database analysis to determine the expression of TIMP3 in cancer immunotherapy. The findings demonstrated that various immunotherapeutic responses were linked to TIMP3 expression (Figure 12B,C). The results showed that TIMP3 expression levels in the CT26 and MC38 model are markedly elevated in anti-PD1 responders following anti-PD1 therapy, but not in non-responders (Figure 12B). Following TNFα and IFNγ cytokine treatment, the expression of TIMP3 was elevated in both CT26 and MC38 models. Consequently, TIMP3 expression is linked to the tumor microenvironment and immune cell infiltration in response to immunotherapy.

## 3. Discussion

Colorectal cancer (CRC) ranks third globally among the most prevalent cancers and remains a leading cause of cancer-related deaths [32]. Understanding immune infiltration in colorectal cancer has been an increasingly prevalent area of research [33]. The rapid development of bioinformatics in recent years has enabled the collection of data from huge sample sizes, which has provided researchers with novel perspectives and approaches for studying and exploring tumors.

Studies reveal that TIMP3 (24-kDa glycoprotein), a member of the TIMP family of proteins, acts as a potent tumor suppressor by preventing tumor angiogenesis, invasion, and metastasis. Notably, TIMP3 distinguishes itself within the TIMP family as the only member that can bind strongly to the extracellular matrix, making it a crucial protein in the control of metastasis [28]. TIMP3 expression is correlated with malignant tendencies in a variety of cancer types, and it is predictive of survival outcomes in breast and hepatocellular carcinoma [34,35]. TIMP3 silencing in tumors is consistently associated with a poor prognosis. Moreover, TIMP3 was shown to induce apoptosis and inhibit cell proliferation [35,36]. These functions collectively establish TIMP3 as an inhibitor in diverse cancers, including CRC.

Our study represents a pioneering effort in integrating data from various sources, including transcriptome, mutation, mRNA, protein, and immune infiltration expression datasets of TIMP3 in CRC. Using multiple databases, such as GEPIA, UALCAN, GeneMANIA, KM Plotter, TIMER, TISIDB, cBioPortal, TIMER, LinkedOmics, TISMO, ScTIME, and TISCH2, we observed notable variation in the expression of TIMP3 within CRC. In particular, TIMP3 expression was lower in COAD tissues than in normal tissues, and a significant correlation emerged between the clinical stages of COAD patients and TIMP3 expression. These findings imply that TIMP3 is a tumor suppressor and plays an important role in suppressing the development, progress, and metastasis of CRC. The multidimensional analysis, encompassing diverse data types, enhances our understanding of the intricate role that TIMP3 plays in CRC pathogenesis. In this study, significant downregulation of TIMP3 was detected in several cancer types, such as lung adenocarcinoma (LUAD), kidney chromophobe (KICH), lung squamous cell carcinoma (LUSC), and uterine carcinosarcoma (UCS) tissues, in addition to CRC.

Analysis of the top 50 interactors of TIMP3 coupled with a protein–protein interaction network revealed that these genes are predominantly connected to extracellular structures and functions and play a substantial role in angiogenesis. Through GO and KEGG enrichment analyses using the GeneMANIA (software version 3.5.2) program and LinkedOmics (http://linkedomics.org/admin.php, accessed on 4 March 2025), we discerned that the metastatic process of colorectal cancer is characterized by complex network functional alterations. In-depth analysis of the top 50 interacting genes using LinkedOmics showed that TIMP3 was directly implicated in the ECM receptor interaction, focal adhesion, protein digestion and absorption, and amoebiasis pathways. Notably, genes associated with TIMP3 were significantly linked to biological processes such as angiogenesis, transmembrane protein transfer, the serine/threonine kinase signaling pathway, amoeboid-type cell migration, and heart morphogenesis, representing enriched terms in this intricate biological landscape. Understanding the complexity of these interactions offers valuable insights into the potential therapeutic implications and prognostic significance of targeting TIMP3 in the context of CRC progression and/or prognosis.

The prevalence, development, and spread of cancer are intricately associated with the extent of immune infiltration in the tumor microenvironment [37]. In recent years, research concerning TME has been crucial for accelerating the development of novel treatment options for cancer, such as immune checkpoint inhibitors, which have demonstrated efficacy in solid tumor types [38,39]. The TME plays an important role in tumor growth, survival, and the ability to metastasize [40]. Within a complicated tumor microenvironment, tumor cells coexist. The broad array of various cells, compounds, and biological factors that surround tumor cells determines the tumor microenvironment [41,42]. The initiation, development, and metastasis of neoplasms are closely associated with tumor-infiltrating immune cells, which are key components of the tumor microenvironment. These immune cells contribute to immunosuppression, hinder anticancer immunity, and support tumor progression [39,40]. For instance, tumor-associated macrophages (TAMs) can polarize into classically activated M1 macrophages, which exert tumoricidal effects, or alternatively into activated M2 macrophages, which are typically immunosuppressive and support tumor progression. Studies have demonstrated that macrophages within the tumor microenvironment (TME) often differentiate into tumor-associated macrophages (TAMs), thereby promoting tumor progression, invasion, and metastasis [43]. In our investigation, we observed a positive correlation between TIMP3 expression and macrophage infiltration in patients with colorectal tumors, as evident from the data analysis using the TIMER and GEPIA programs. Furthermore, TIMP3 showed substantial and favorable relationships with gene markers linked to tumor-associated macrophages (TAMs) and macrophages. Thus, TIMP3 may affect prognosis by controlling TAMs in the tumor microenvironment, which could potentially extend the lives of patients. Further in-depth research has suggested that tumor-infiltrating dendritic cells (DCs) tend to foster immunosuppression and tolerance within the tumor microenvironment, rather than promoting anticancer immunity [43,44]. These findings contribute to our understanding of intricate interactions within the TME, providing valuable insights into the potential impact of TIMP3 on immune regulation in CRC. Our study presents a relationship between TIMP3 expression and different TIICs or immunomodulators, signifying a close connection between TIMP3 and immune infiltration in CRC patients. Recent data suggest that the interaction between circulating tumor cells and neutrophils may promote distant metastasis of tumor cells [45]. Furthermore, by encouraging the epithelial–mesenchymal transition (EMT) process, macrophages have been demonstrated to improve tumor cell migration and invasion [46].

Establishing connections between these genes not only enhances our understanding, but also facilitates the identification of potential multi-gene-based therapeutic strategies for clinicians. Data from cBioPortal revealed that sequence changes in TIMP3 are infrequent events in CRC. In 2850 analyzed CRC samples, the combined frequency of structural variants, copy number alterations, and point mutations was merely 1%. The limited occurrence of TIMP3 sequence and copy number alterations in CRC strongly suggests that they are unlikely to serve as primary drivers of the observed under-expression of TIMP3. Consequently, our investigation leans towards the likelihood of an alternative molecular mechanism contributing to this phenomenon.

CD4^+^ T cells are a crucial part of the TME. CD4^+^ T cells may differentiate into a variety of phenotypically unique subgroups, including Th1, Th2, and Th17 cells [47]. Our data analyses revealed a positive correlation between TIMP3 expression and the infiltration levels of B cells, CD8^+^ T cells, CD4^+^ T cells, macrophages, neutrophils, and dendritic cells in CRC. Moreover, there was a noteworthy association between increased TIMP3 expression and various immune-related factors, including chemokines, chemokine receptors, immunostimulators, and immunoinhibitors. These findings support a potential immunological role of TIMP3 in CRC and suggest that reduced TIMP3 expression, through modulation of the immune microenvironment, may influence CRC outcomes.

Chemokines, classified as chemotactic cytokines, play a crucial role in mediating immune cell trafficking, cell proliferation, migration, invasion, and angiogenesis [48,49]. Our results showed that the expression of TIMP3 correlated positively with chemokines such as CCL21, CCL2, CCL12, CCL18, CCL14, CCL7, and CCL19 and negatively with CCL15. In parallel, chemokine receptors, including CCR6, showed negative correlations, while CCR1, CCR8, CCR2, CXCR1, CCR5, CCR4, CXCR4, and CXCR5 showed strong positive connections with TIMP3 expression. This intricate interplay between TIMP3 and chemokines, along with their receptors, suggests a substantial influence on both pro- and anti-tumorigenic immune responses. Chemokines and their receptors play a pivotal role in shaping the tumor microenvironment, highlighting their significance in orchestrating immune dynamics within the tumor milieu [50,51]. Immunomodulatory drugs are currently under development for various conditions and have received approval for certain tumors, such as multiple myeloma [52,53]. In our exploration, we identified a spectrum of immunoinhibitory and immunostimulatory molecules that exhibit a positive correlation with TIMP3. TGFβ1 exhibits the maximum positive correlation, followed by CSF1R, HAVCR1, KDR, TGFβR1, ADORA, and TIGIT. On the other hand, all immune stimulators were found to exhibit a positive correlation amongst all the immune-stimulatory molecules studied.

We also investigated the function of TIMP3 in the TME and discovered that its expression was favorably connected with immune cells such CD8 cells, fibroblasts, and macrophages. Gene set enrichment analysis (GSEA) further revealed that TIMP3 is associated with multiple immune-related pathways. These findings imply that TIMP3 could be involved in the CRC TME. The expression levels of various genes associated with immunity in a variety of tumor types were correlated with TIMP3 expression. Our validation results from the TIDE database also demonstrated that TIMP3 had a greater predictive value for response to immunotherapy. This suggests that, for the majority of tumor types, TIMP3 has superior predictive potential for immunotherapy response, as many modules showed AUC values of more than 0.5. According to current research, our findings could offer a fresh approach to immunotherapy biomarker creation and mining.

Diverse immune cell types play distinct roles in the growth of tumors. Although M1 macrophages, NK cells, and CD8^+^ T cells are known to be anticancer cells, Tregs, myeloid-derived suppressor cells (MDSCs), cancer-associated fibroblasts (CAFs), and M2 macrophages are commonly thought to being tumor-accelerative cells. We found that M1 macrophages and TIMP3 are consistently positively correlated, which is thought to be significant for the production of an inhibitory CRC TME. The findings indicate that M1-associated markers are more abundant in the TIMP3^high^ group, suggesting that TIMP3 may hinder the development of CRC by activating the antitumor properties of M1 macrophages. Additionally, four single-cell sequencing results obtained from TISCH2 demonstrated a strong correlation between TIMP3 and macrophages.

TIMP3 has the potential to be a therapeutic target and an indicator of tumor susceptibility to numerous anticancer drugs, in addition to being a biomarker for predicting the development of cancer [54,55]. CRC patients with lower TIMP3 expression levels exhibited poorer overall survival rates. Taken together, our findings provide novel insights into TIMP3 as a prognostic marker and drug target for CRC. Thus, TIMP3 expression measurements in cancer patients could find applications in clinical assessments, offering valuable insights into disease assessment and prognosis forecasting. Anticipating further research on TIMP3’s biological function in the tumor immune milieu, we envision a clearer understanding of its role in cancer prognosis.

### Limitations

This study has several limitations that must be addressed in future research. Our conclusions are based entirely on bioinformatics analyses of public databases, and experimental validation is necessary to confirm these findings. Due to the absence of clinical cohorts of colorectal cancer (CRC) patients undergoing immunotherapy, TIMP3’s role in predicting immunological responses in CRC could not be evaluated. While functional enrichment analysis and PPI network construction provided useful insights into TIMP3-associated genes, these methods may not fully capture the complexity of gene functions in specific biological contexts. Further experimental studies, such as immunohistochemistry and western blotting, are needed to analyze the protein expression levels of relevant immune-related genes.

We have identified genes associated with differential TIMP3 expression levels in CRC that could offer similar clinical value, due to their comparable roles in CRC. However, the clinical application of immune-related gene signatures must be validated in larger prospective trials. TIMP3 and related gene expression can be quantified using q-PCR in CRC cell lines and patient samples, and protein expression can be analyzed by immunohistochemistry and western blotting to establish its association with clinical and pathological characteristics. Gene knockout or overexpression experiments, pathway analysis, and mechanistic studies can provide further insights in the molecular pathways and cellular processes involved in CRC pathogenesis. Furthermore, while the correlation between TIMP3 expression levels and clinical outcomes like overall survival and disease stage provides valuable predictive insights, it is important to note that these conclusions are based on correlation studies and retrospective data, and hence require experimental validation.

Ultimately, expanding our research regarding TIMP3’s molecular mechanisms in influencing cancer patient prognoses offers potential for improving tumor diagnosis and treatment. TIMP3 emerges as a promising biomarker with potential applications in cancer research and clinical practice, making it a novel target for further investigation.

## 4. Materials and Methods

### 4.1. Ethics Statement

The present investigation made use of publicly accessible databases and excluded any research or experiments involving humans or animals. Therefore, ethical approval was not necessary for this research.

### 4.2. Acquisition of Patient Data

All patient clinical data and messenger RNA (mRNA) information was obtained from the Genotype-Tissue Expression (GTEx) (https://commonfund.nih.gov/GTEx (accessed on 4 March 2025)) and The Cancer Genome Atlas (TCGA) (https://www.cancer.gov/ccg/research/genome-sequencing/tcga) databases accessed on 4 March 2025.

### 4.3. GEPIA

Gene expression profiling interactive analysis (GEPIA) is a data-driven analytic tool based on TCGA and GTEx data that includes RNA sequence expression data from 9736 cancers and 8587 normal tissue samples [56]. The GEPIA database (http://gepia.cancer-pku.cn/index.html, accessed on 4 March 2025) is a publicly accessible web server for tumor and normal gene expression profiling and interactive analysis based on information from the TCGA database and GTEx. The present investigation employed GEPIA single-gene analysis to examine variations in the mRNA expression of TIMP3 between tumor and normal tissues. All plotting features in GEPIA are developed using the R (version 3.3.2) and Perl (version 5.22.1) programs. To construct the box plot, a single-gene analysis module was adopted, with a P cut-off value of 0.05 and a log2FC cut-off value of 1, as well as the option “Match TCGA normal value”. The data (counts) were converted to transcripts per million (TPM) format and normalized using log2 (TPM + 1). Log2 (TPM + 1) data were utilized for logarithmic scaling to generate box plots.

### 4.4. UALCAN

A useful website for online analysis and mining of cancer data is UALCAN (http://ualcan.path.uab.edu/index.html, accessed on 4 March 2025), which is mostly based on pertinent cancer data from the TCGA database [57]. The current study examined the relationship between clinicopathological characteristics and the protein expression of TIMP3 in CRC tissues using UALCAN. Student’s t-test was employed to compare variations in transcriptional expression. * *p*  <  0.05, ** *p*  <  0.01, and *** *p*  <  0.001 were considered statistically significant.

### 4.5. LinkedOmics

A multi-omics data analysis of 32 TCGA cancer types is available in LinkedOmics [58] (http://linkedomics.org/admin.php, accessed on 6 March 2025 and 21 July 2025). LinkedOmics was used in this study for biological investigations of TIMP family kinase target enrichment. In the COAD READ datasets, GSEA was used for 500 simulations and at least three genes. The significance criterion was set at *p* ≤ 0.05 for the Spearman correlation test. Pearson’s correlation coefficient was used to quantitatively assess the genes associated with TIMP3 in CRC, and heat maps and volcano plots were used to display the results.

### 4.6. GeneMANIA

GeneMANIA (http://genemania.org, accessed on 7 March 2025) offers predictions based on genetic, DNA, and protein–protein interactions, as well as reactions, pathways, and protein domains of associated genes [59]. Through analysis of a substantial amount of background association data and the gene association functions in the list, one can expand the provided gene list. In this study, gene association and related gene exploration were carried out using the Gene MANIA server.

### 4.7. TIMER

The tumor immune estimation resource (TIMER) version 2.0 database (http://timer.comp-genomics.org, accessed on 8 April 2025) includes 10,009 samples from 23 different cancer types in the TCGA. It is a comprehensive online resource for systematically assessing the clinical relevance and differential gene correlation of tumor–immune transcripts [60]. This study employed the gene module to assess the relationship between immune cell infiltration and TIMP3 levels.

To ensure accurate assessment of immune correlations, we utilized the immunedeconv R package, which integrates six advanced algorithms: TIMER, XCELL, CIBERSORT, EPIC, MCPCOUNTER, and QUANTISEQ. R programming was not used as a separate tool; instead, the analysis was performed directly through the GENE module feature of TIMER 2.0 database (https://cistrome.shinyapps.io/timer, accessed on 8 April 2025). Upon adjusting for purity, scatterplots were generated to display the relationship between TIMP3 expression and cancer-associated cell infiltration. Furthermore, the “Gene_DE” module of the TIMER 2.0 database (https://cistrome.shinyapps.io/timer, accessed on 8 April 2025). was used to assess variations in gene expression levels between distinct tumor tissues and their neighboring normal tissues.

The Shiny web framework in R was used to create TIMER 3.0, a publicly available web server for the scientific community [61]. The four immune deconvolution methods that constitute it include immune, immunotherapy, estimation, and exploration. Users can explore the way estimated immune infiltrates relate to gene expression, somatic mutations, somatic copy number changes (sCNAs), and clinical outcomes across tumor cohorts using the four modules that make up the immunological component. CIBERSORT evaluates the proportions of 22 immune cells based on RNA-seq data by applying a deconvolution algorithm. Through the exploration, we tried to understand important relationships inside tumors.

### 4.8. TISIDB

A web portal for tumor and immune system interaction, the TISIDB database (http://cis.hku.hk/TISIDB, accessed on 9 April 2025) incorporates many heterogeneous data types. In addition to multiple resources for immunological data collected from seven public databases, it connects to 988 documented immune-related anti-tumor genes, high-throughput screening methods, molecular profiling, and para-cancerous multi-omics data. In the present investigation, the TISIDB dataset was used to investigate the relationship between TIMP3 expression and immune modulators, including immunosuppressors, immunostimulants, chemokines, and receptors [62].

### 4.9. cBioPortal

Cancer genomics datasets are available on the Cancer Genomics Portal (http://www.cbioportal.org/, accessed on 10 April 2025), which is also used to investigate genetic alterations in different types of cancer [63]. The Cancer Type Summary module obtained all TCGA tumors with the chosen mutation type, copy number alteration (CNA), and alteration frequency. The “Mutations” module allows the mutation site information of TIMP3 to be shown in a three-dimensional (3D) or schematic diagram of the protein structure. Moreover, we acquired information about the overall, progression-free, disease-specific, and disease-free survival differences between CRC tumor cases with and without genetically altered TIMP3 using “cBioportal”. Additionally, log-rank *p* values were obtained for Kaplan–Meier plots.

### 4.10. TISCH2

To illustrate the TIMP3 expression profile at the single-cell level in various datasets of CRC cancer types, we created a heatmap using the TISCH2 database (http://tisch.comp-genomics.org/, accessed on 15 April 2025) [64]. We generated a two-dimensional heatmap representation of this high-dimensional data using a Uniform Manifold Approximation and Projection (UMAP) approach. To create a thorough scRNA reference library and precisely determine the cellular makeup of every area on the 10xVisium plate, we employed deconvolution analysis for the spatial transcriptome data. A single-cell dataset that has been previously published (GSE166555) was utilized to ascertain how TIMP3 affected myeloid-derived cells and lymphoid-derived cells.

### 4.11. SCTIME

The SCTIME database [65] (http://sctime.sklehabc.com/, accessed on 15 April 2025) was used to identify the network of interactions between immune cells and cancerous cells. The SCTIME database was also used to examine the relationships between DC and macrophage abundances and TIMP3 expression in CRC cells.

### 4.12. CAMOIP

Comprehensive Analysis on Multi-Omics of Immunotherapy in Pan-cancer (CAMOIP) database (https://www.camoip.net/, accessed on 16 April 2025) provides users with the ability to explore the manner in which immune cells, immune genes, and immune-related scores have changed over time. The immune cell fraction is calculated using CIBERSORT, MCPcounter, EPIC, quanTIseq, and IPS [66]. The CIBERSORT databases was used to examine differences in expression between immune-related genes in different groups, i.e., TIMP3^High^ and TIMP3^Low^ samples. The CAMOIP database was utilized to analyze and visualize the differences in mutant genes between the groups with high and low TIMP3 expression.

### 4.13. TISMO and TIDE

Syngeneic mouse models and tumor models are available in the Tumor Immune Syngeneic MOuse (TISMO) database (http://tismo.cistrome.org, accessed on 17 April 2025) [67] to analyze tumor immunity and immunotherapy response in 23 different cancer types. Since it contains more than 2000 high-quality RNA sequencing samples from cancer cell lines or syngeneic mouse tumor models, the TISMO database is frequently used to forecast immunotherapy response based on individual gene expressions. In syngeneic colon cancer cell lines expressing the TIMP3 gene, interactions with colon cancer and immune regulation were examined in connection with cytokine therapy and immune checkpoint blockade (ICB) response. This efficacy was subsequent compared with several well-known indicators obtained from the TIDE (Tumor Immune Dysfunction and Exclusion) database (http://tide.dfci.harvard.edu/, accessed on 17 April 2025) [68], such as TIDE, Merck 18, B clonality, T clonality, IFNG, CD8, CD274, TMB, and MSI score.

### 4.14. Statistics

The relationship between TIMP3 expression and immunostimulators, immunoinhibitors, chemokines, receptors, and immunological infiltration levels was examined using Spearman’s correlation coefficient. *p* ≤ 0.05 was considered significant. To assess the relationship between TIMP3 expression in the TIMER, GEPIA, and UALCAN datasets, Spearman’s correlation analysis was employed. * *p* ≤  0.05, ** *p*  ≤  0.01, and *** *p* ≤  0.001 were considered statistically significant. Most statistical analyses were automatically conducted using the default settings, if not otherwise indicated, with the detailed dataset information and following the statistical methods outlined in their respective databases.

## 5. Conclusions

In conclusion, our research highlights the significant differential expression of TIMP3, particularly in colorectal adenocarcinoma, where it emerges as an influential factor in prognosis and clinical traits. Its expression is intricately linked with immune infiltration in CRC, suggesting a regulatory role in the immune microenvironment and contributing to its prognostic impact. As a promising biomarker, TIMP3 holds the potential for predicting outcomes in CRC cases with immune infiltration. With all aspects considered, this work bridges the gap between immune response and gene expression in colorectal cancer (CRC), expanding our knowledge of TIMP3 as a tumor suppressor and immune landscape regulator with potential implications for immunotherapeutic targeting.

Our analysis underscores TIMP3’s immunoregulatory properties, positioning it as a predictive indicator for patient prognosis. TIMP3 influences immune cell infiltration, including T cells and macrophages, making it a potential target or co-target in immunotherapeutic strategies. It can serve as prognostic biomarker, as TIMP3 expression levels have been correlated with patient survival across various cancers, including glioblastoma, breast cancer, colorectal cancer, and thyroid cancer. Low TIMP3 expression is frequently associated with poor prognosis, increased tumor aggressiveness, and metastasis. It may also be used as a predictive marker for drug sensitivity in colorectal cancer.

## Data Availability

The authors declare that the data supporting the findings of this study are addressed within the article.

## Abbreviations

COAD	Colon adenocarcinoma
CRC	Colorectal cancer
DFS	Disease-free survival
ECM	Extracellular matrix
FDR	False detection rate
GTEx	Genotype–Tissue Expression
HR	Hazard ratio
KICH	Kidney chromophobe
LUAD	Lung adenocarcinoma
LUSC	Lung squamous cell carcinoma
MMP	Matrix metalloproteinase
OS	Overall survival
PFS	Progression-free survival
READ	Rectal adenocarcinoma
RFS	Relapse-free survival
TAM	Tumor-associated macrophages
TCGA	The Cancer Genome Atlas
TIICs	Tumor-infiltrating immune cells
TIMP3	Tissue inhibitor of matrix metalloproteinase
TME	Tumor microenvironment
TPM	Transcripts per million
UCS	Uterine carcinoma
VEGF	Vascular endothelial growth factor

## References

[1] Leslie A., Steele R.J.C. (2002). Management of colorectal cancer. Postgrad. Med. J..

[2] Bray F., Laversanne M., Sung H., Ferlay J., Siegel R.L., Soerjomataram I., Jemal A. (2024). Global cancer statistics 2022: GLOBOCAN estimates of incidence and mortality worldwide for 36 cancers in 185 countries. CA Cancer J. Clin..

[3] Sung H., Ferlay J., Siegel R.L., Laversanne M., Soerjomataram I., Jemal A., Bray F. (2021). Global Cancer Statistics 2020: GLOBOCAN Estimates of Incidence and Mortality Worldwide for 36 Cancers in 185 Countries. CA Cancer J. Clin..

[4] Siegel R.L., Miller K.D., Wagle N.S., Jemal A. (2023). Cancer statistics, 2023. CA Cancer J. Clin..

[5] Renkonen-Sinisalo L., Aarnio M., Mecklin J.P., Järvinen H.J. (2000). Surveillance improves survival of colorectal cancer in patients with hereditary nonpolyposis colorectal cancer. Cancer Detect. Prev..

[6] Siegel R.L., Giaquinto A.N., Jemal A. (2024). Cancer statistics, 2024. CA Cancer J. Clin..

[7] Dekker E., Tanis P.J., Vleugels J.L.A., Kasi P.M., Wallace M.B. (2019). Colorectal cancer. Lancet.

[8] Jayasinghe M., Prathiraja O., Caldera D., Jena R., Coffie-Pierre J.A., Silva M.S., Siddiqui O.S. (2023). Colon Cancer Screening Methods: 2023 Update. Cureus.

[9] Brenner H., Stock C., Hoffmeister M. (2014). Effect of screening sigmoidoscopy and screening colonoscopy on colorectal cancer incidence and mortality: Systematic review and meta-analysis of randomised controlled trials and observational studies. BMJ.

[10] Kim H.M., Kim T.I. (2024). Screening and surveillance for hereditary colorectal cancer. Intest. Res..

[11] Vogel J.D., Felder S.I., Bhama A.R., Hawkins A.T., Langenfeld S.J., Shaffer V.O., Thorsen A.J., Weiser M.R., Chang G.J., Lightner A.L. (2022). The American Society of Colon and Rectal Surgeons Clinical Practice Guidelines for the Management of Colon Cancer. Dis. Colon Rectum.

[12] Fornieles G., Núñez M.I., Expósito J. (2023). Matrix Metalloproteinases and Their Inhibitors as Potential Prognostic Biomarkers in Head and Neck Cancer after Radiotherapy. Int. J. Mol. Sci..

[13] Jiang Y., Goldberg I.D., Shi Y.E. (2002). Complex roles of tissue inhibitors of metalloproteinases in cancer. Oncogene.

[14] Su C.-W., Lin C.-W., Yang W.-E., Yang S.-F. (2019). TIMP-3 as a therapeutic target for cancer. Ther. Adv. Med. Oncol..

[15] Lin H., Zhang Y., Wang H., Xu D., Meng X., Shao Y., Lin C., Ye Y., Qian H., Wang S. (2012). Tissue inhibitor of metalloproteinases-3 transfer suppresses malignant behaviors of colorectal cancer cells. Cancer Gene Ther..

[16] Cruz-Munoz W., Sanchez O.H., Di Grappa M., English J.L., Hill R.P., Khokha R. (2006). Enhanced metastatic dissemination to multiple organs by melanoma and lymphoma cells in timp-3−/− mice. Oncogene.

[17] English W.R., Ireland-Zecchini H., Baker A.H., Littlewood T.D., Bennett M.R., Murphy G., Pintus G. (2018). Tissue Inhibitor of Metalloproteinase–3 (TIMP-3) induces FAS dependent apoptosis in human vascular smooth muscle cells. PLoS ONE.

[18] Qi J.H., Ebrahem Q., Ali M., Cutler A., Bell B., Prayson N., Sears J., Knauper V., Murphy G., Anand-Apte B. (2013). Tissue Inhibitor of Metalloproteinases-3 Peptides Inhibit Angiogenesis and Choroidal Neovascularization in Mice. PLoS ONE.

[19] Anania M.C., Sensi M., Radaelli E., Miranda C., Vizioli M.G., Pagliardini S., Favini E., Cleris L., Supino R., Formelli F. (2011). TIMP3 regulates migration, invasion and in vivo tumorigenicity of thyroid tumor cells. Oncogene.

[20] Guan Z., Zhang J., Song S., Dai D. (2013). Promoter methylation and expression of TIMP3 gene in gastric cancer. Diagn. Pathol..

[21] Liu H.-Q., Song S., Wang J.-H., Zhang S.-L. (2011). Expression of MMP-3 and TIMP-3 in gastric cancer tissue and its clinical significance. Oncol. Lett..

[22] Dindarloo M.M., Fendereski A., Kashi Z., -Charati J.Y. (2024). Long-Term Effect of TIMP3 Gene Expression on Thyroid Cancer: A Cure Model Analysis. Asian Pac. J. Cancer Prev..

[23] Zhang W., Zhang P., Wang X., Lin Y., Xu H., Mao R., Zhu S., Lin T., Cai J., Lin J. (2024). SORBS2-Mediated inhibition of malignant behaviors in esophageal squamous cell carcinoma through TIMP3. Int. Immunopharmacol..

[24] Han J., Jing Y., Han F., Sun P. (2021). Comprehensive analysis of expression, prognosis and immune infiltration for TIMPs in glioblastoma. BMC Neurol..

[25] Mazzoni M., Todoerti K., Agnelli L., Minna E., Pagliardini S., Di Marco T., Borrello M.G., Neri A., Greco A. (2022). Transcriptomic landscape of TIMP3 oncosuppressor activity in thyroid carcinoma. Cancer Cell Int..

[26] Su C.-W., Su B.-F., Chiang W.-L., Yang S.-F., Chen M.-K., Lin C.-W. (2017). Plasma levels of the tissue inhibitor matrix metalloproteinase-3 as a potential biomarker in oral cancer progression. Int. J. Med. Sci..

[27] Han X.-G., Mo H.-M., Liu X.-Q., Li Y., Du L., Qiao H., Fan Q.-M., Zhao J., Zhang S.-H., Tang T.-T. (2018). TIMP3 Overexpression Improves the Sensitivity of Osteosarcoma to Cisplatin by Reducing IL-6 Production. Front. Genet..

[28] Huang H.-L., Liu Y.-M., Sung T.-Y., Huang T.-C., Cheng Y.-W., Liou J.-P., Pan S.-L. (2019). TIMP3 expression associates with prognosis in colorectal cancer and its novel arylsulfonamide inducer, MPT0B390, inhibits tumor growth, metastasis and angiogenesis. Theranostics.

[29] Lao V., Grady W. (2012). The Role of Timp3 in the Pathogenesis of Colorectal Cancer and Timp3 Promoter Methylation as a Potential Predictive Marker for Egfr Inhibitor Therapy. J. Surg. Res..

[30] Hinshaw D.C., Shevde L.A. (2019). The Tumor Microenvironment Innately Modulates Cancer Progression. Cancer Res..

[31] Ong S., Tan Y., Beretta O., Jiang D., Yeap W., Tai J.J.Y., Wong W., Yang H., Schwarz H., Lim K. (2012). Macrophages in human colorectal cancer are pro-inflammatory and prime T cells towards an anti-tumour type-1 inflammatory response. Eur. J. Immunol..

[32] Haggar F.A., Boushey R.P. (2009). Colorectal Cancer Epidemiology: Incidence, Mortality, Survival, and Risk Factors. Clin. Colon Rectal Surg..

[33] Picard E., Verschoor C.P., Ma G.W., Pawelec G. (2020). Relationships Between Immune Landscapes, Genetic Subtypes and Responses to Immunotherapy in Colorectal Cancer. Front. Immunol..

[34] Gu X., Fu M., Ding Y., Ni H., Zhang W., Zhu Y., Tang X., Xiong L., Li J., Qiu L. (2014). TIMP-3 expression associates with malignant behaviors and predicts favorable survival in HCC. PLoS ONE.

[35] Mylona E., Magkou C., Giannopoulou I., Agrogiannis G., Markaki S., Keramopoulos A., Nakopoulou L. (2006). Expression of tissue inhibitor of matrix metalloproteinases (TIMP)-3 protein in invasive breast carcinoma: Relation to tumor phenotype and clinical outcome. Breast Cancer Res..

[36] Jackson H.W., Defamie V., Waterhouse P., Khokha R. (2017). TIMPs: Versatile extracellular regulators in cancer. Nat. Rev. Cancer.

[37] Kao K.-C., Vilbois S., Tsai C.-H., Ho P.-C. (2022). Metabolic communication in the tumour–immune microenvironment. Nat. Cell Biol..

[38] Waldman A.D., Fritz J.M., Lenardo M.J. (2020). A guide to cancer immunotherapy: From T cell basic science to clinical practice. Nat. Rev. Immunol..

[39] Ma W., Xue R., Zhu Z., Farrukh H., Song W., Li T., Zheng L., Pan C.-X. (2023). Increasing cure rates of solid tumors by immune checkpoint inhibitors. Exp. Hematol. Oncol..

[40] Genova C., Dellepiane C., Carrega P., Sommariva S., Ferlazzo G., Pronzato P., Gangemi R., Filaci G., Coco S., Croce M. (2022). Therapeutic Implications of Tumor Microenvironment in Lung Cancer: Focus on Immune Checkpoint Blockade. Front. Immunol..

[41] de Visser K.E., Joyce J.A. (2023). The evolving tumor microenvironment: From cancer initiation to metastatic outgrowth. Cancer Cell.

[42] Baker A.H., George S.J., Zaltsman A.B., Murphy G., Newby A.C. (1999). Inhibition of invasion and induction of apoptotic cell death of cancer cell lines by overexpression of TIMP-3. Br. J. Cancer.

[43] Steven A., Seliger B. (2018). The Role of Immune Escape and Immune Cell Infiltration in Breast Cancer. Breast Care.

[44] Ngambenjawong C., Gustafson H.H., Pun S.H. (2017). Progress in tumor-associated macrophage (TAM)-targeted therapeutics. Adv. Drug Deliv. Rev..

[45] Mizuno R., Kawada K., Itatani Y., Ogawa R., Kiyasu Y., Sakai Y. (2019). The Role of Tumor-Associated Neutrophils in Colorectal Cancer. Int. J. Mol. Sci..

[46] Liaghat M., Ferdousmakan S., Mortazavi S.H., Yahyazadeh S., Irani A., Banihashemi S., Asl F.S.S., Akbari A., Farzam F., Aziziyan F. (2024). The impact of epithelial-mesenchymal transition (EMT) induced by metabolic processes and intracellular signaling pathways on chemo-resistance, metastasis, and recurrence in solid tumors. Cell Commun. Signal..

[47] Speiser D.E., Chijioke O., Schaeuble K., Münz C. (2023). CD4+ T cells in cancer. Nat. Cancer.

[48] Wei C., Yang C., Wang S., Shi D., Zhang C., Lin X., Liu Q., Dou R., Xiong B. (2019). Crosstalk between cancer cells and tumor associated macrophages is required for mesenchymal circulating tumor cell-mediated colorectal cancer metastasis. Mol. Cancer.

[49] Braoudaki M., Ahmad M.S., Mustafov D., Seriah S., Siddiqui M.N., Siddiqui S.S. (2022). Chemokines and chemokine receptors in colorectal cancer; multifarious roles and clinical impact. Semin. Cancer Biol..

[50] Ozga A.J., Chow M.T., Luster A.D. (2021). Chemokines and the immune response to cancer. Immunity.

[51] Bule P., Aguiar S.I., Aires-Da-Silva F., Dias J.N.R. (2021). Chemokine-Directed Tumor Microenvironment Modulation in Cancer Immunotherapy. Int. J. Mol. Sci..

[52] Davis L.N., Sherbenou D.W. (2021). Emerging Therapeutic Strategies to Overcome Drug Resistance in Multiple Myeloma. Cancers.

[53] Maes K., Mondino A., Lasarte J.J., Agirre X., Vanderkerken K., Prosper F., Breckpot K. (2021). Epigenetic Modifiers: Anti-Neoplastic Drugs with Immunomodulating Potential. Front. Immunol..

[54] Yuan L.-Q., Liu Y.-S., Luo X.-H., Guo L.-J., Xie H., Lu Y., Wu X.-P., Liao E.-Y. (2008). Recombinant tissue metalloproteinase inhibitor-3 protein induces apoptosis of murine osteoblast MC3T3-E1. Amino Acids.

[55] Span P.N., Lindberg R.L., Manders P., Tjan-Heijnen V.C., Heuvel J.J., Beex L.V., Sweep C. (2004). Tissue inhibitors of metalloproteinase expression in human breast cancer: TIMP-3 is associated with adjuvant endocrine therapy success. J. Pathol..

[56] Tang Z., Li C., Kang B., Gao G., Li C., Zhang Z. (2017). GEPIA: A web server for cancer and normal gene expression profiling and interactive analyses. Nucleic Acids Res..

[57] Chandrashekar D.S., Karthikeyan S.K., Korla P.K., Patel H., Shovon A.R., Athar M., Netto G.J., Qin Z.S., Kumar S., Manne U. (2022). UALCAN: An update to the integrated cancer data analysis platform. Neoplasia.

[58] Vasaikar S.V., Straub P., Wang J., Zhang B. (2018). LinkedOmics: Analyzing multi-omics data within and across 32 cancer types. Nucleic Acids Res..

[59] Warde-Farley D., Donaldson S.L., Comes O., Zuberi K., Badrawi R., Chao P., Franz M., Grouios C., Kazi F., Lopes C.T. (2010). The GeneMANIA prediction server: Biological network integration for gene prioritization and predicting gene function. Nucleic Acids Res..

[60] Li T., Fan J., Wang B., Traugh N., Chen Q., Liu J.S., Li B., Liu X.S. (2017). TIMER: A Web Server for Comprehensive Analysis of Tumor-Infiltrating Immune Cells. Cancer Res..

[61] Cui H., Zhao G., Lu Y., Zuo S., Duan D., Luo X., Zhao H., Li J., Zeng Z., Chen Q. (2025). TIMER3: An enhanced resource for tumor immune analysis. Nucleic Acids Res..

[62] Ru B., Wong C.N., Tong Y., Zhong J.Y., Zhong S.S.W., Wu W.C., Chu K.C., Wong C.Y., Lau C.Y., Chen I. (2019). TISIDB: An integrated repository portal for tumor–immune system interactions. Bioinformatics.

[63] Cerami E., Gao J., Dogrusoz U., Gross B.E., Sumer S.O., Aksoy B.A., Jacobsen A., Byrne C.J., Heuer M.L., Larsson E. (2012). The cBio Cancer Genomics Portal: An Open Platform for Exploring Multidimensional Cancer Genomics Data. Cancer Discov..

[64] Han Y., Wang Y., Dong X., Sun D., Liu Z., Yue J., Wang H., Li T., Wang C. (2023). TISCH2: Expanded datasets and new tools for single-cell transcriptome analyses of the tumor microenvironment. Nucleic Acids Res..

[65] Hong F., Meng Q., Zhang W., Zheng R., Li X., Cheng T., Hu D., Gao X. (2021). Single-Cell Analysis of the Pan-Cancer Immune Microenvironment and scTIME Portal. Cancer Immunol. Res..

[66] Lin A., Qi C., Wei T., Li M., Cheng Q., Liu Z., Luo P., Zhang J. (2022). CAMOIP: A web server for comprehensive analysis on multi-omics of immunotherapy in pan-cancer. Brief. Bioinform..

[67] Zeng Z., Wong C.J., Yang L., Ouardaoui N., Li D., Zhang W., Gu S., Zhang Y., Liu Y., Wang X. (2022). TISMO: Syngeneic mouse tumor database to model tumor immunity and immunotherapy response. Nucleic Acids Res..

[68] Jiang P., Gu S., Pan D., Fu J., Sahu A., Hu X., Li Z., Traugh N., Bu X., Li B. (2018). Signatures of T cell dysfunction and exclusion predict cancer immunotherapy response. Nat. Med..

