# Correction: Bhola et al. An Integrative Bioinformatics Approach to Investigating TIMP3 and Immune Cell Infiltration: Prognostic and Clinicopathological Implications. *Int. J. Mol. Sci.* 2025, *26*, 8867

**DOI:** 10.3390/ijms27062688

**Published:** 2026-03-16

**Authors:** Neelam Bhola, Chanchal Bareja, Amit K. Jaiswal, Daman Saluja

**Affiliations:** 1 Dr. B.R. Ambedkar Center for Biomedical Research, University of Delhi, Delhi 110007, India; neelambhola@gmail.com (N.B.); chanchal1862@gmail.com (C.B.); 2 School of Food Science and Environmental Health, Faculty of Sciences and Health, Technological University Dublin—City Campus, Central Quad, Grangegorman, D07 H6K8 Dublin, Ireland; amit.jaiswal@tudublin.ie; 3 Department of Allied and Basic Sciences, Shri Guru Gobind Singh Tricentenary University, Gurugram 122505, India

In the published manuscript [1], there was an error in the authorship. Dr. Chanchal Bareja was not included as an author in the original publication. Authors have confirmed her equal contribution with Dr. Amit K. Jaiswal. The corrected Author Contributions statement appears here. The authors state that the scientific conclusions are unaffected. This correction was approved by the Academic Editor. The original publication has also been updated.
